# Possibilities, limits and particularities of plastic surgery treatment in HIV-associated lipodystrophy

**DOI:** 10.1186/1471-2334-14-S7-O37

**Published:** 2014-10-15

**Authors:** Dana Jianu, Anca Streinu-Cercel, Maria Filipescu, Ștefan Jianu, Mihaela Vartic, Oltjon Cobani, Adrian Streinu-Cercel

**Affiliations:** 1 Proestetica Medical Center, Bucharest, Romania; 2 Carol Davila University of Medicine and Pharmacy, Bucharest, Romania; 3 National Institute for Infectious Diseases "Prof. Dr. Matei Balş", Bucharest, Romania

## Background

Given the particularities of the Romanian HIV cohort, an important number of patients have received treatment with first-generation antiretroviral drugs and have developed an associated lipodystrophy. Nowadays HIV-infected patients can benefit from less invasive surgical treatments for lipodystrophy, to ameliorate their psychosomatic status.

## Case report

We present 2 cases of patients with HIV-associated lipodystrophy. These patients underwent specific modern “closed” plastic surgery corrections: liposuction and lipo-redistribution. The patients were evaluated with a follow-up of 10 years (the first case) and respectively 6 months (the second case). In each case the possibilities and limits of these treatments were evaluated.

## Conclusion

Treatment of HIV infection should be effective but it should also lead to an improvement in the quality of life. A multidisciplinary approach – infectious diseases physicians, plastic surgeons, anesthesiologists, histopathologists, psychologists etc. – can contribute to a psychosomatic improvement.

